# Microbial Profiling of Biltong Processing Using Culture-Dependent and Culture-Independent Microbiome Analysis

**DOI:** 10.3390/foods12040844

**Published:** 2023-02-16

**Authors:** Caitlin Karolenko, Udaya DeSilva, Peter M. Muriana

**Affiliations:** 1 Robert M. Kerr Food and Agricultural Products Center, Oklahoma State University, Stillwater, OK 74078, USA; 2 Department of Animal and Food Sciences, Oklahoma State University, Stillwater, OK 74078, USA

**Keywords:** biltong, microbiome, air-dried beef, *Carnobacterium* sp., *Latilactobacillus* sp.

## Abstract

Biltong is a South African air-dried beef product that does not have a heat lethality step, but rather relies on marinade chemistry (low pH from vinegar, ~2% salt, spices/pepper) in combination with drying at ambient temperature and low humidity to achieve microbial reduction during processing. Culture-dependent and culture-independent microbiome methodologies were used to determine the changes in the microbial community at each step during biltong processing through 8 days of drying. Culture-dependent analysis was conducted using agar-based methods to recover viable bacteria from each step in the biltong process that were identified with 16S rRNA PCR, sequencing, and BLAST searching of the NCBI nucleotide database. DNA was extracted from samples taken from the laboratory meat processing environment, biltong marinade, and beef samples at three stages of processing (post-marinade, day 4, and day 8). In all, 87 samples collected from two biltong trials with beef obtained from each of three separate meat processors (n = six trials) were amplified, sequenced with Illumina HiSeq, and evaluated with bioinformatic analysis for a culture-independent approach. Both culture-dependent and independent methodologies show a more diverse population of bacteria present on the vacuum-packaged chilled raw beef that reduces in diversity during biltong processing. The main genera present after processing were identified as *Latilactobacillus* sp., *Lactococcus* sp., and *Carnobacterium* sp. The high prevalence of these organisms is consistent with extended cold-storage of vacuum-packaged beef (from packers, to wholesalers, to end users), growth of psychrotrophs at refrigeration temperatures (*Latilactobacillus* sp., *Carnobacterium* sp.), and survival during biltong processing (*Latilactobacillus sakei*). The presence of these organisms on raw beef and their growth during conditions of beef storage appears to ‘front-load’ the raw beef with non-pathogenic organisms that are present at high levels leading into biltong processing. As shown in our prior study on the use of surrogate organisms, *L. sakei* is resistant to the biltong process (i.e., 2-log reduction), whereas *Carnobacterium* sp. demonstrated a 5-log reduction in the process; the recovery of either psychrotroph after biltong processing may be dependent on which was more prevalent on the raw beef. This phenomenon of psychrotrophic bloom during refrigerated storage of raw beef may result in a natural microbial suppression of mesophilic foodborne pathogens that are further reduced during biltong processing and contributes to the safety of this type of air-dried beef.

## 1. Introduction

Biltong is a South African style dried beef product made using lean beef rounds that are sliced, marinated in a mixture of salt, vinegar, and spices, and then dried at ambient temperature and humidity. Since biltong is produced without a heat lethality step, the safety of the product relies on the addition of vinegar and salt in the marinade step and an extended drying period to achieve a low water activity (A_w_) to make the product safe for consumers [1,2]. Biltong manufacturers obtain beef rounds from a variety of meat processors that have been chilled, vacuum-packaged, and stored prior to use in the biltong production process. This ‘wet-aging’ stage takes place between slaughter, sale, and final use during which the meat has remained vacuum-packed in an oxygen-barrier film and stored above freezing temperatures for up to several weeks [3].

Chilled storage and packaging helps to prevent the growth of common foodborne pathogens and slows the rate of meat spoilage [4,5]. However, it also leads to the multiplication of psychrophiles and psychrotrophs on the raw beef. The type of packaging used (oxygen impermeable/permeable, gas flushed) together with various intrinsic (pH, A_w_, nutrient content) and extrinsic factors (temperature, relative humidity, process conditions, environmental contamination) can select for different types of bacteria that colonize the meat surface [6,7,8].

Bacteria found in the processing environment (slaughter, fabrication, further processing) also add to the total microbiota on the meat. This in combination with any contributing bacteria found in added processing ingredients (i.e., marinade ingredients) results in the initial microbiota of the food product [9,10]. The initial microbiota can then change as processing conditions change (i.e., temperature, drying). Other studies have investigated the contribution of the environment on chilled, vacuum-packaged beef as well as the changes in microbial communities on dried beef products during processing [11,12,13].

Microbial profiling during biltong manufacturing can be performed to determine whether the process selects for specific organisms. This can be achieved in one of two ways: culture-dependent methodology or culture-independent methodology. Culture-dependent methodology relies on agar-based methods to isolate, identify, and characterize bacteria from the food matrix. While culture-based methodologies are standard in many laboratories and industry, culture-dependent techniques can only detect 0.1% of a complex community, overestimating bacterial species that are culturable while underestimating species that are unculturable or below detection limits [14,15]. Therefore, to understand the extent of all bacteria present, nucleic acid sequencing-based techniques (i.e., culture-independent methods) can be used to understand the complex microbiomes of foods. Previous studies have utilized culture-independent microbiome analysis to investigate the bacterial community of processed meats, including modified atmosphere packaged beef, beef steaks and dry-aged beef from manufacturing facility to final product, including the influence of the facility environment on the microflora of the final product [16,17,18].

The objectives of this study were to identify bacterial populations present at different stages of biltong processing and if they change during processing through culture-independent and culture-dependent microbiome analyses. This change in bacterial population was assessed using duplicate trials of beef obtained from each of three different beef processors to determine the influence different facilities may have on the native vs. final microbiota of biltong beef.

## 2. Materials and Methods

### 2.1. Beef Sources

Beef was obtained from 3 different meat producers and used in biltong trials with dual objectives of both culture-dependent (colony isolation, 16S rRNA identification) and culture-independent microbiome analysis (DNA extraction, 16S gene sequencing, and microbial community analysis). USDA Select grade bottom round beef was sourced from Nebraska Beef (Omaha, NE, USA), Greater Omaha Packing Co, Inc. (Omaha, NE, USA), and High River Angus (JBS USA Food Company, Greeley, CO, USA). Beef from each of these processors was purchased from a local meat processor (Ralph’s Packing Co., Perkins, OK, USA) who obtained the beef from a regional beef broker. The beef rounds were then transported to a cold room at the Robert M. Kerr Food and Agricultural Products Center at Oklahoma State University (FAPC, Stillwater, OK, USA) and stored for 2–3 days at 4 °C before use.

### 2.2. Beef Preparation and Sampling

#### 2.2.1. Preparation of Beef for Biltong Process and Microbiome Analyses

The same beef and biltong process would serve as a source of samples for both culture-dependent and culture-independent microbiome analyses. Initial trials of DNA extraction tests (DNeasy PowerFood Microbial DNA extraction kit, Qiagen, Germantown, MD, USA) with raw untrimmed beef were of low quality due to high lipid content that interfered with DNA extraction buffers [19]. We therefore followed the procedures of Hanlon et al. [20] to massage the vacuum-packaged bags and recover purge to minimize fat recovery. Vacuum-packaged beef bottom rounds were hand-massaged for 60 s to encourage detachment of bacteria from meat pieces with minimal disruption to the fat [20].

Bags were carefully sanitized prior to opening using a sanitized knife, new gloves and sanitized trays and care was taken to minimize introduction of external contamination. After removal of the bottom rounds, 30 mL of purge liquid was taken to represent bacteria on the surface of the beef, distributed into two separate 15 mL sterile conical centrifuge tubes, and placed in a refrigerator for 30 min to allow the lipid content at the surface to solidify. An additional 2 mL of purge sample was collected for enumeration purposes. Following refrigeration, the liquid portion from each tube was removed from the solidified lipid layer and placed in new sterile 15 mL conical tubes (two 15 mL tubes per sample). The tubes were centrifuged at 4280 g for 20 min at 4 °C. The supernatant fractions were discarded, and 1 mL of cold sterile molecular-grade water was added to resuspend the pellet. The entire volume was then transferred to the second tube and used to resuspend the second pellet, thus combining the two tubes into one sample. The combined resuspended pellets were then transferred to a 2 mL DNA-free sterile microcentrifuge tube to start DNA extraction for culture-independent analysis.

#### 2.2.2. Biltong Beef Processing, Marination, and Drying

Each beef round was trimmed of fat, sliced lengthwise, and cut to approximately 5.1 cm wide × 1.9 cm thick × 7.6 cm long beef rectangles, and held on covered trays overnight at 5 °C. One round (~15 lbs) from each of the commercial packing plants was sufficient for one biltong trial. Beef used in these trials were never frozen and used within 2–3 days of receipt.

Biltong processing was conducted as described previously by Karolenko et al. [1,21], but without bacterial inoculation. Individual beef pieces were placed in plastic baskets and dipped in sterile water in stainless steel vessels to replicate water rinse treatments or antimicrobial dips to enhance microbial reduction that is often used by biltong processors. The beef pieces were placed in the water for 30 s, after which the basket was removed and excess liquid allowed to drain for 60 s. The beef pieces were then placed into chilled stainless steel tumbling vessels containing a biltong marinade consisting of 2.2% salt, 0.8% black pepper, 1.1% coarse ground coriander, and 4% red wine vinegar (100-grain, 10% acetic acid) in relation to the total meat weight. Beef pieces were vacuum-tumbled (15 inches Hg; Biro VTS-43 Marblehead, OH, USA) for 30 min and then hung to dry in a humidity-controlled oven (Hotpack, Model #435315, Warminster, PA, USA) at 55% relative humidity and 24.9 °C (75 °F) for 8 days.

During each duplicate trial run from each of the 3 beef suppliers (n = 6 per sampling period per supplier), samples were collected at each sampling time point (raw beef/purge, beef after marinade, and beef after 4 and 8 days of drying). Beef samples were placed in a sterile Whirl-Pak filter stomaching bag (Nasco, Fort Atkinson, WI, USA) with 50 mL of sterile water and stomached to resuspend attached bacteria. DNA extraction from raw beef gave low yields of DNA, presumably because of interference from fat (similar extractions from post-marinaded raw beef provided acceptable yields of DNA). Because of this problem, we used purge from the package as representative of the microbiota present on raw beef from the supplier as per Hanlon et al. [20].

All samplings were performed in triplicate at each stage of duplicate biltong trials from each of the 3 beef processors (n = 6/sampling point/beef supplier).

#### 2.2.3. Environmental and Marinade Sample Preparation for Microbiome Analysis

Environmental samples were also collected from surfaces that the meat would have contacted during fabrication and processing. Sterile premoistened sponge swabs (Sponge Sticks, 3M, St. Paul, MN, USA) were used to sample the cutting knife (both sides of the blade), cutting tray (plastic, 360 cm^2^) and gloves of the person trimming the beef. The same sponge was used to swab all three surfaces. Swabbing of the environmental surfaces was performed in triplicate for each trial. A clean tray, knife, and fresh gloves were used for trimming and cutting separate rounds from each processor if multiple beef bottom rounds were processed. Following swabbing, 25 mL of sterile water was added to the sample bag and hand massaged for 60 s. The resulting liquid was collected in sterile 15 mL conical tubes. Tubes were centrifuged at 4280 g for 20 min at 4 °C. The supernatant was discarded, and 1 mL of cold sterile water was added to one of the two tubes per sample to resuspend the pellet. The entire volume was then transferred to the second tube and used to resuspend the second pellet, thus combining the two tubes into one sample. The combined resuspended pellet was then transferred to a 2 mL DNA-free sterile microcentrifuge tube to start DNA extraction for culture-independent analysis.

Three separate marinade samples were made for each of the 2 trials performed for each of the 3 beef processors tested. Each marinade was formulated based on 100 g of meat (the average amount of beef per individual biltong sample). Sterile water (8.3 mL) was added to the marinade, and samples were hand stomached for 60 s at high setting and transferred to a sterile 15 mL conical tube. Tubes were centrifuged at 4280 g for 20 min at 4 °C. The supernatant was discarded, and 1 mL of cold sterile water was added to resuspend the pellet. The combined resuspended pellet was then transferred to a 2 mL DNA-free sterile microcentrifuge tube to start DNA extraction for culture-independent analysis.

### 2.3. Culture-Dependent Analysis

#### 2.3.1. Bacterial Enumeration

Microbial enumeration was evaluated by total viable aerobic bacterial counts. Serial 10-fold dilutions were made by transferring 1 mL of each sample from the stomacher bags into 9 mL sterile 0.1% buffered peptone water (BPW, BD Bacto) tubes. Dilutions were then surface plated (0.1 mL) on tryptic soy agar (TSA) plates (BD Bacto; 1.5% agar) in duplicate and incubated for 48 h at 30 °C before being counted. 

#### 2.3.2. Microbial Profiling (16S PCR, Sequencing, and Identification of Isolates)

Following incubation and enumeration, five bacterial isolates were collected from the countable petri plate dilution obtained at each timepoint (raw beef, post-marination, day 4 and 8 of drying), and for each duplicate trial for each of the 3 processors (i.e., a total of 10 isolates/timepoint/beef processor). An effort was made to select phenotypically different colonies if present. Isolates were streaked onto TSA for further purification and then single colony isolates were inoculated into tryptic soy broth (TSB) and allowed to grow for 24 h at 30 °C. Bacterial cells were harvested (1 mL) by centrifugation and washed three times in 0.1 M Tris Buffer (pH 8.0) and lysed by the bead beating method [22] using sterile, acid-washed glass beads (425–600 μm; Sigma) to extract the DNA. The resulting DNA template was then used for 16S rRNA polymerase chain reaction (PCR) to amplify the 16S-related DNA. Amplification for each isolate was performed with two separate reactions each with a forward and reverse primer. The first reaction utilized the primers 7F (5′-RAGAGTTTGATCHTGGCTCAG-3′) and 928R (5′-CCCCGTCAATTCHTTTGA-3′) and the second reaction was performed using the primers 759F (5′-CAGGATTAGATACCCTGGTAGTCC-3′) and 1541R (5′-AAGGAGGTGATCCARCCGC-3′). Following amplification, the resulting PCR amplimers were cleaned using the GenCatch Advanced PCR Extraction Kit (Epoch Life Sciences; Missouri City, TX, USA) per the manufacturer’s procedures. The cleaned up amplimers were sent to the Core DNA Sequencing Facility at Oklahoma State University (Stillwater, OK, USA) for sequencing in both the forward and reverse direction for each template. Sequences were aligned using Molecular Evolutionary Genetic Analysis (MEGA X software, version 10.2.6 [23]), and then identified by using Standard Nucleotide BLAST (blast.ncbi.nlm.nih.gov) to compare our sequences with those in the NCBI 16S rRNA gene sequence database.

#### 2.3.3. Phylogenetic Relationship

The relationship between the identified isolates was derived using phylogenetic tree analysis of the data using the Maximum Likelihood method in MEGA X software [23]. The evolutionary analyses were conducted in MEGA X software. Initial trees were automatically generated using the Neighbor-Joining method and pairwise distances were computed for the variant trees with the Maximum Composite Likelihood approach. The topology with the superior likelihood value was selected.

### 2.4. Culture-Independent Analysis

#### 2.4.1. DNA Extraction

The resuspended pellets from Section 2.2.3 were extracted with the DNeasy PowerFood Microbial DNA extraction kit and protocol (Qiagen). All samples were eluted using 100 μL elution buffer and then quantified using a NanoDrop-1000 spectrophotometer (Thermo Fisher Scientific, Waltham, MA, USA; [24]). Extracted DNA was stored at −20 °C until needed. Samples were stored in 1.5 mL Eppendorf tubes wrapped in Parafilm^Tm^ M (Thermo Fisher), placed in a sample box wrapped in a plastic bag, and placed in a Styrofoam shipping box with 10 pounds of dry ice. The box was then shipped by overnight carrier to Novogene Co. Sample Receiving (Sacramento, CA, USA) for further amplification, sequencing, and analysis.

#### 2.4.2. 16S rRNA Sequencing

From the extractions, 96 samples (30 from Greater Omaha; 36 from Nebraska Beef; 30 from High River-JBS) were submitted to Novogene (for 16S rRNA sequencing and analysis. A mock community made up equal proportions of *Escherichia coli* ATCC BAA 1427, *Enterococcus faecium* 201224-016, and *Pediococcus acidilactici* ATCC 8042 was used as a positive control for comparison. Per the company’s protocols, the V3-V4 regions of the 16S bacterial rRNA gene were amplified using primers 341F (5′-CCTAYGGGRBGGASCAG-3′) and 806R (5′-GGACTACNNGGGTATCTAAT-3′). Amplicons were sequenced on Illumina NovaSeq 6000 paired-end platform to generate ~470 base pair paired-end reads. Paired-end reads were then merged using FLASH (V1.2.7) creating raw tags (30 K). Raw tags were quality filtered to obtain clean tags via QIIME (V.1.7.0) software. Tags were then compared with a reference database (SILVA138 database) using UCHIME to detect chimera sequences. Any chimera sequences found were removed, obtaining effective tags used for bioinformatic analysis. Analyses were initially performed with the inclusion of non-bacterial data but were also conducted with the mitochondria and chloroplast related sequences removed for comparison.

#### 2.4.3. Bioinformatic Analysis

Bioinformatic analysis was conducted at Novogene Co. Sequence analysis was performed using Uparse software (Uparse V.7.0.1090) using all effective tags. For OTU analysis, Novogene uses >97% similarity as the minimum threshold for OTU analysis of sequence similarity while current studies have recommended higher species identity threshold levels of at least 99% [25]. Species annotation at each taxonomic rank (threshold 0.1~1) was performed in QIIME against the SSU rRNA database of SILVA138 database. Further phylogenetic relationships of OTUs were obtained using MUSCLE (Version 3.8.31). Relative abundances were normalized using a standard of sequence number corresponding to the sample with the least sequences.

### 2.5. Growth Assay of Isolates Obtained from Biltong Process

Select bacterial isolates collected from various meat suppliers, including *C. divergens* GO R1B, *C. gallinarum* NB R1C, *C. gallinarum* NB R2A, and *L. sakei* GO R2D, were tested in comparison to mesophilic bacteria, including *Enterococcus faecium 201224-016* and *E. coli* ATCC BAA-1427, for growth at 5 °C and 30 °C. All strains were transferred twice from frozen stock and finally 50 uL was inoculated into 5 mL TSB (*Carnobacterium*, *E. faecium*, *E. coli*) or MRS broth (*L. sakei* GO R2D) in spectrophotometer tubes. The separate sets of inoculated pre-chilled or pre-warmed media tubes were incubated at both 5 °C (for 7 days) and 30 °C (for 24 h). Un-inoculated media tubes (TSB, MRS) for use as ‘blanks’ were incubated along with the inoculated tubes. Absorbance (590 nm) of each tube was obtained using a Spectronic-20D+ spectrophotometer (model 333183, Thermo Fisher Scientific, Waltham, MA, USA). To maintain the incubation temperature for the short duration outside of the incubator during readings, each test tube rack was kept in a metal bin filled with an ice slurry to submerge the tubes to the level of the broth. Absorbance readings were obtained every hour for the samples incubated at 30 °C over the course of 10 h. Samples incubated at 5 °C were read after inoculation (0 h), after 10 h, and then every 24 h thereafter for 7 days.

## 3. Results and Discussion

### 3.1. Culture-Dependent Microbiome Analysis

#### 3.1.1. Bacteria Identified via Culture-Dependent Methodology

A total of 30 isolates were collected from three sources of raw beef (10 from each processor). An additional 31 isolates were collected from the marinaded, dried beef (11 isolates were collected from Greater Omaha; 10 isolates each from Nebraska Beef and High River-JBS). The identification of the bacteria isolated during biltong processing via culture-dependent methodology is compiled in Table 1, Table 2 and Table 3. It is generally accepted by taxonomists that % identity scores of ≥97% and ≥99% for 16S rRNA gene sequences are sufficient to identify organisms down to genus and species level, respectively [26,27,28].

**Figure 1:**
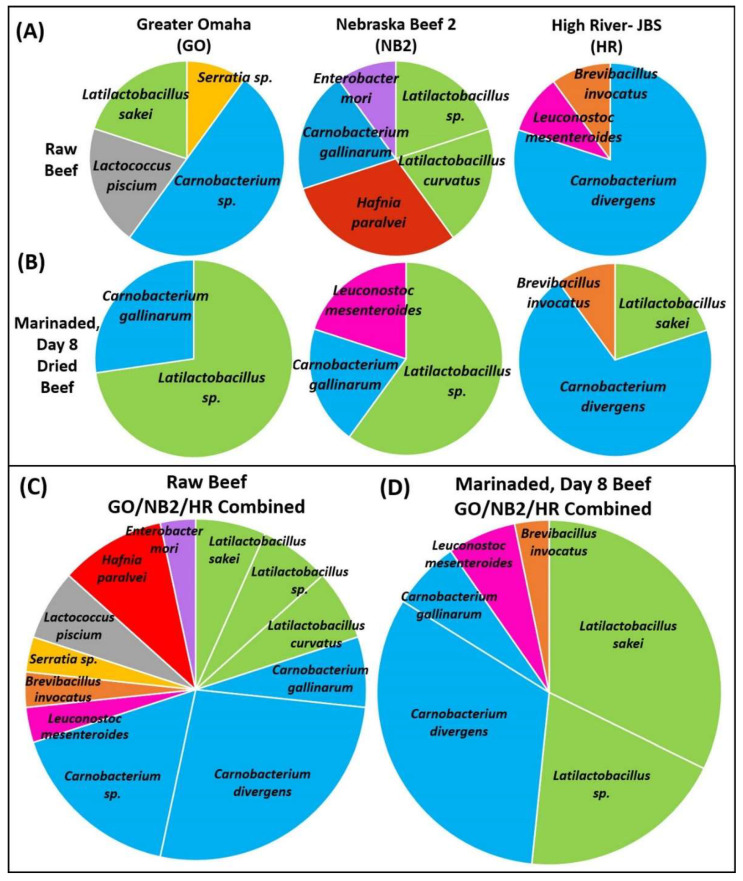
Culture-dependent microbiome analysis. Bacteria isolated and identified using 16S rRNA PCR and sequencing from duplicate biltong trials with beef from each of the 3 processors: (**A**) raw beef and (**B**) biltong that had been marinaded and dried for 8 days. Pie chart analysis of all isolates from the three processors combined from (**C**) raw beef and (**D**) day 8 dried beef.

A general trend among the identified isolates from all three processors is a greater variation in the bacterial species found on the raw beef and then a decrease in diversity after the beef had been marinated and dried for eight days (Figure 1). The remaining bacteria on the beef after the biltong process were predominantly members of *Carnobacterium* or *Latilactobacillus* as determined by culture-dependent isolation.

Culture-dependent microbiome analysis demonstrated a greater diversity of organisms recovered from raw beef yet showing overlapping similarity between the three different sources of beef. Raw beef isolates from Greater Omaha Inc. were identified as *Carnobacterium gallinarum* (50%), *Lactococcus piscium* (20%), *Latilactobacillus* sp. (20%), and *Serratia* sp. (10%) (Figure 1A). The raw beef isolates from Nebraska Beef included *Latilactobacillus curvatus* (20%), others identified only as *Latilactobacillus* sp. (20%), *C. gallinarum* (20%), and *Enterobacter mori* (10%) (Figure 1A). Raw beef isolates from High River-JBS were primarily *C. divergens* (80%), *L. mesenteroides* (10%), and *Brevibacillus inovatus* (10%) (Figure 1A).

After processing by salt/vinegar/spice marination followed by 8 days of drying, *Latilactobacillus* sp. (*L. sakei*; 72.7%) and *Carnobacterium sp.* (*C. gallinarum*; 27.3%) accounted for 100% of isolates from Greater Omaha; *Latilactobacillus* sp. (60%), *C. gallinarum* (20%) and *Leuconostoc mesenteroides* (20%) from Nebraska Beef; and *Carnobacterium* sp. (*C. divergens*, 70%), *Latilactobacillus* sp. (*L. sakei*, 20%), and *B. invocatus* (10%) from High River-JBS (Figure 1B). These data are also presented as the aggregate of the raw vs. processed isolates from the combined sources showing that *Carnobacterium* sp., and *Latilactobacillus* sp. dominate the post-process microbiota (Figure 1C,D). These results are consistent with other studies in which spoilage-related bacteria found on raw, chilled beef stored in vacuum-packaged products included *Pseudomonas*, *Carnobacterium*, *Rahnella*, *Serratia*, *Hafnia*, and *Enterobacter* [8,29,30,31]. In a comparative genomic analysis of *Carnobacterium* sp., the identification of a cell surface secretome capable of heme uptake, biopolymer hydrolysis, and biopolymer adhesins, renders some *Carnobacterium* sp. well adapted for survival in the animal gut [32]. Similar studies of *L. sakei* suggest it is a transient inhabitant of animal intestinal tracts that is also well suited for attachment, growth, and survival on raw meat surfaces after slaughter [33,34].

#### 3.1.2. Impact of Processing on Culture-Dependent Microbiome

**Figure 2:**
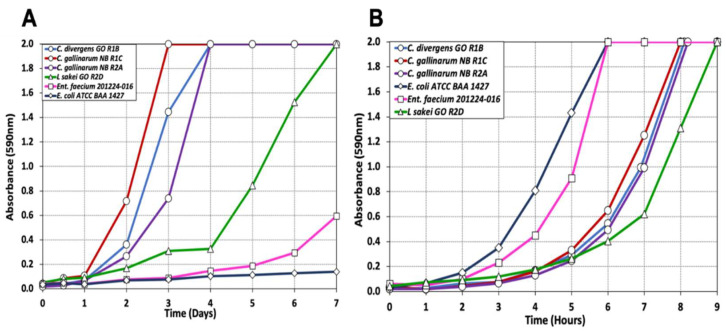
Spectrophotometric growth assay of bacteria isolated from marinated and dried biltong beef (*C. divergens* GO R1B; *C. gallinarum* NB R1C; *C. gallinarum* NB R2A; *L. sakei* GO R2D) compared to known mesophilic bacteria (*E. faecium* 201224-016; *E. coli* BAA ATCC 1427) at incubation temperatures (**A**) 5 °C and (**B**) 30 °C. Absorbance (at 590 nm) of each culture was measured every hour at 30 °C and every 24 h at 5 °C.

The data show that *Carnobacterium* sp. and *Latilactobacillus* sp. are the predominant bacteria on raw beef from three processors used to source beef in our study. This can be attributed to an extended period of refrigerated, vacuum-packaged storage, also known as ‘wet-aging’ or ‘vacuum aging’, of raw beef prior to their use in biltong processing [35,36]. Both *Carnobacterium* sp. and *Latilactobacillus* sp. are known psychrotrophic bacteria that can grow under refrigerated conditions during wet-aging of vacuum-packaged beef [30,37,38]. We compared the growth of three strains of *Carnobacterium* (*C. divergens* GO R1B, *C. gallinarum* NB R1C, NB R2A) and one strain of *L. sakei* GO R2D isolated during our biltong process along with several organisms commonly associated with animals (*E. faecium*, *E. coli*). During spectrophotometric growth assays at 5 °C, all three strains of *Carnobacterium* reached their maximum level of growth in 3–4 days, *L. sakei* reached maximum growth at 7 days, whereas the mesophilic *E. faecium* and *E. coli* lagged significantly behind (Figure 2A). At moderate temperatures (30 °C), the mesophiles grew faster than the psychrotrophs as one might expect (Figure 2B). The data show growth in separate isolated nutrient environments (test tubes), and likely if they were mixed on the same meat surface, the psychrotrophs could further outcompete the mesophiles by using up the available nutrient supply to where the mesophiles would be even less competitive because available growth nutrients were already depleted.

The ability of *Carnobacterium* sp. and *Latilactobacillus* sp. to grow under refrigerated conditions underscores their ability to out compete mesophilic bacteria and demonstrates how they can quickly dominate the microbial community to become ephemeral spoilage organisms leading into various types of raw beef processing [39,40]. It would not be unusual for vacuum-packaged beef to be held under refrigerated storage for several weeks from fabrication through end use, thereby establishing a healthy psychrotrophic microbiota as evidenced by the culture-dependent data (Table 1, Table 2 and Table 3; Figure 1) and that shown in Figure 2A. These organisms may be considered protective cultures because of their ability to outcompete potential pathogenic bacteria on fresh/raw beef and their potential to make antimicrobial peptides (bacteriocins) [37,41]. Depending on the intended use of fresh packaged-beef (as raw beef vs. processed beef products), these organisms could become spoilage organisms if their growth is not interrupted by processing, such as during biltong manufacture [42,43,44].

**Figure 3:**
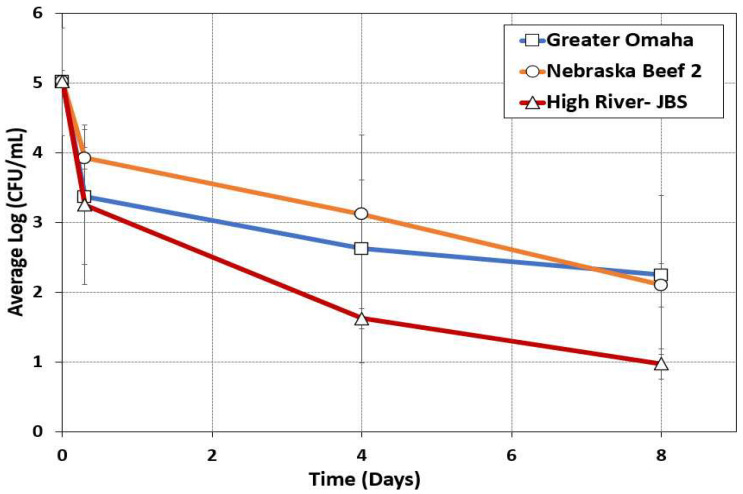
Aerobic plate count (APC) enumeration of bacteria recovered from biltong during duplicate processing trials of beef obtained from each of three beef producers (Greater Omaha Beef Co., Nebraska Beef, and High River/JBS). Surviving bacteria were enumerated at four different timepoints during biltong processing, including: raw beef, beef after marination, and marinaded beef dried for 4 and 8 days at 24.9 °C (75 °F) and 55% RH. Samples were surface plated on TSA and incubated for 48 h at 30 °C prior to enumeration. The graph curves were averaged from duplicate trials and sampled in triplicate at each timepoint (n = 6).

In prior studies with pathogen-inoculated beef, we followed the decline of a pathogenic inoculum throughout the biltong process [1,2,21]. In the current study, we enumerated APCs of the indigenous microbiota of the meat during the biltong process. Bacteria were enumerated from duplicate trials performed on beef from each of three processors at each step of the biltong process, including the raw beef prior to processing, after vacuum-tumbling marination in salt, spice, and vinegar, and again after four and eight days of drying (Figure 3).

The initial reduction after the marinade step is due to exposure of surface bacteria to low pH (vinegar) and high salt conditions in the marinade. USDA-FSIS prefers validation studies to be performed with ‘acid-adapted’ cultures to ensure inoculum bacteria are not overly sensitive to acidic treatments during processing [45]. It is not clear if use of acid-adapted cultures works as expected, because it is also known that many bacteria demonstrate cross-reactive responses to stresses after surviving exposure to other kinds of stress [46,47,48]. The remaining bacteria were then further reduced during desiccation whereby up to 60% moisture loss is incurred and the initial 2.2% salt concentration may reach upwards of >4% salt. The salt, along with low humidity drying, results in A_w_ levels below 0.85 A_w_ by the end of the biltong process. Similar reductions have been observed in previous biltong validation studies [21]. The beef from Nebraska Beef and Greater Omaha had the least overall reduction in APC counts during biltong processing, just shy of an overall process reduction of 2.8–2.9-log. This correlates to the culture-dependent data that show the dominant bacteria in the day 8 marinaded beef was *L. sakei*. Alternatively, the beef from High River-JBS had a total reduction of just over 4-log, with the dominant bacteria at the end of processing being *C. divergens*. These data correlate with those obtained using *L. sakei* and *Carnobacterium* sp. inoculated beef as potential surrogate bacteria during biltong validation studies, whereby *Carnobacterium* sp. had >5-log process reduction while *L. sakei* only achieved a 1.8–2.0-log reduction by day 8 of drying [49].

#### 3.1.3. Phylogenetic Relationship among Isolates Obtained from Biltong Processing

**Figure 4:**
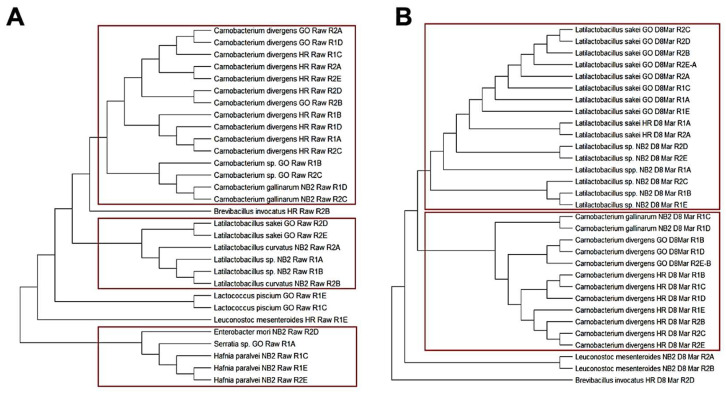
Dendrograms of all isolates obtained and identified from the (**A**) raw beef and (**B**) marinaded, day 8 dried beef from all three tested beef processors: Greater Omaha (GO), Nebraska Beef (NB2), and High River—JBS (HR). The phylogenic trees were constructed using the Maximum Likelihood method with pairwise distances estimated using the Maximum Composite Likelihood approach. Evolutionary analyses were conducted in MEGA X software. The red square highlight clades of importance related to the identified isolates and their processor of origin.

Further analysis was performed with the isolates to determine their relationship in respect to the origin of the beef (Figure 4). Sequence alignment and hierarchical cluster analysis is a useful tool for phylogenetic analysis [50]. Phylogenetic analysis shows the predominance of *Carnobacterium* spp. Among isolates obtained from raw beef (Figure 4A), whereas *L. sakei* represent a majority after 8 days of drying (Figure 4B). The prevalence of *Carnobacterium* sp. pre-process (Figure 4A) is likely due to their faster growth rate (than *L. sakei*) during refrigerated storage of raw beef (i.e., Figure 2), whereas the prevalence of *L. sakei*/*Latilactobacillus* sp. post-process (Figure 4B) reflects the ability of this organism to survive the biltong process (i.e., only a ~2 log reduction) better than *Carnobacterium* sp. (i.e., ~5 log reduction) [49]. It would be interesting to know if these bacteria are contributed to raw beef by contact contamination in their processing plants or transferred directly from the animal during slaughter. Determination of this would require access to, and sampling of, the beef carcasses and their respective manufacturing facilities.

### 3.2. Culture-Independent Microbiome Analysis

#### 3.2.1. Bacterial Richness and Diversity in Biltong Processing

A total of 8,285,608 raw tags (3,047,152 tags from Greater Omaha samples; 3,085,216 from Nebraska Beef samples; 2,153,240 tags from High River-JBS) from 87 samples were sequenced using the Illumina platform. Following quality filtering and removal of non-bacterial sequences, a total of 7,952,944 clean tags remained. Additional chimera filtration steps resulted in a remaining 6,993,542 sequences used for further analysis. The environmental samples from the High River-JBS sample set did not have sufficient yield of extracted nucleic acid and were therefore excluded from further library preparation and sequencing.

**Figure 5:**
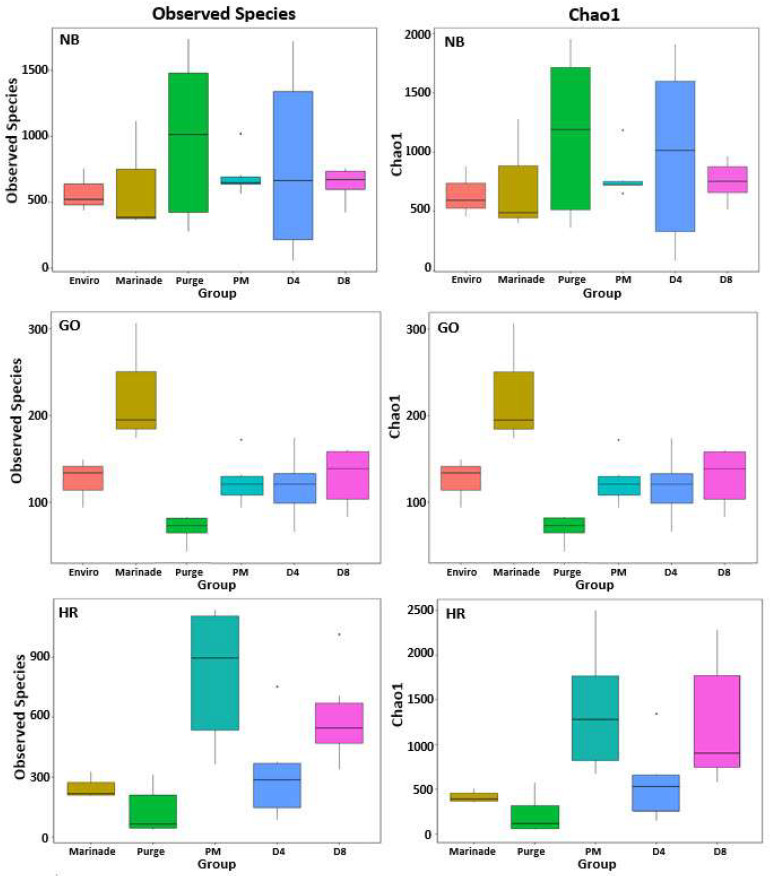
Box plot of differences between groups of Observed Species and Chao1 from different beef processors (Nebraska Beef, NB; Greater Omaha, GO; High River, HR). Wilcox rank sum test and Tukey test were used to analyze the differences and significance (*p* < 0.05) in species diversity between groups.

Alpha diversity analysis including observed species and Chao1 was conducted to determine differences in diversity between each timepoint of the biltong process from each processor separately. The alpha diversities are represented in the form of boxplots (Figure 5).

Based on observed species data, the raw beef/purge samples from Greater Omaha and High River-JBS had the lowest diversity in all the steps of the biltong processing that were sampled (Figure 5). Following the marinade step, the marinated beef was significantly higher (*p* < 0.05) compared to the raw meat/purge samples in the Greater Omaha and High River-JBS samples. After marination, there was no significant difference in diversity between the post-marinated (PM), day 4 (D4) and day 8 (D8) samples from Greater Omaha. The D4 bacterial community was significantly different (*p* < 0.05) from PM and D8 on the meat from High River-JBS. There was no significant difference in any of the indices with the samples from Nebraska Beef. The decrease in diversity at day 4 with the beef from High River was unexpected. Both chemical (enzyme mediated) and physical (beads) lysing techniques were included in the DNA extraction process to achieve maximum yield and improve accuracy of bacterial community structure (i.e., not favoring Gram-negative bacteria since they are easier to lyse) [51]. However, the amount of DNA extracted from the day 4 samples from High River-JBS was lower compared to the post-marinated and day 8 samples from the same processor which could have contributed to a decrease in microbial diversity [52].

#### 3.2.2. Changes in the Microbial Community during Processing

**Figure 6:**
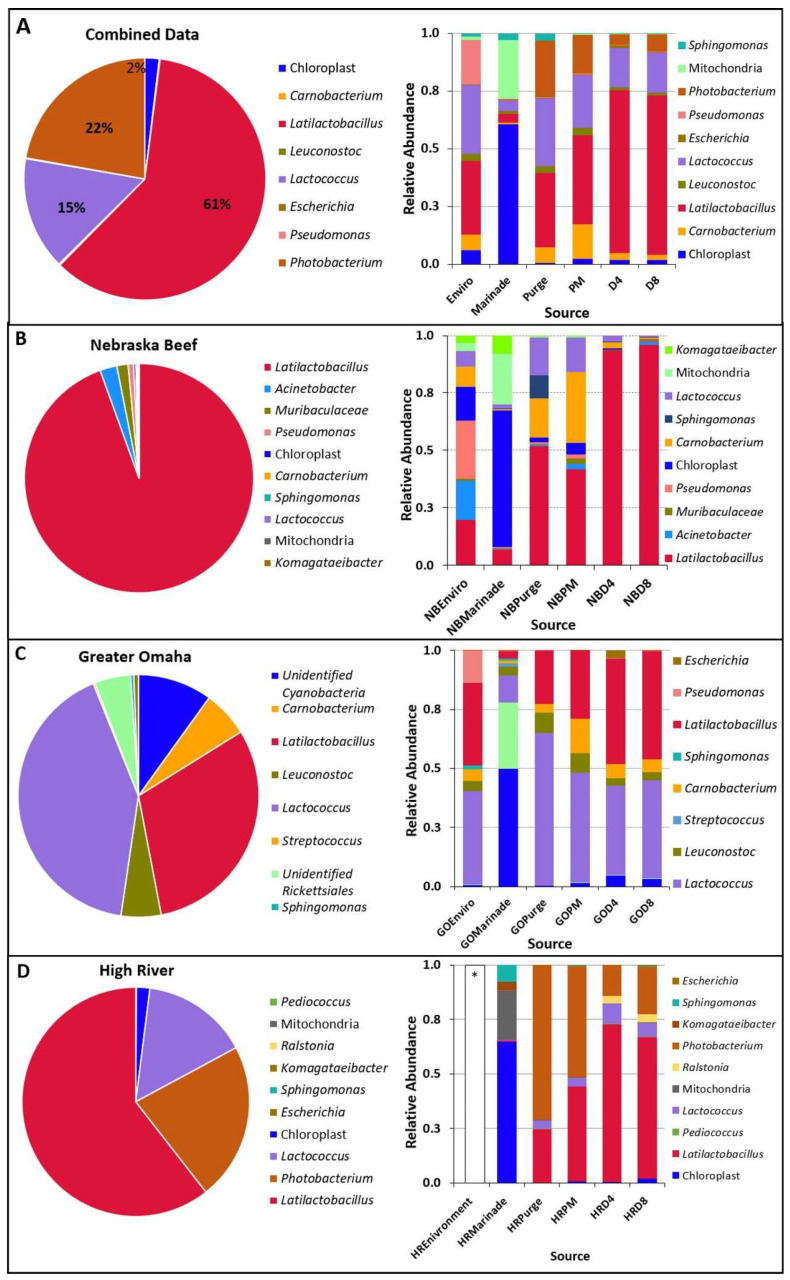
Relative abundance of total identified DNA from across all processing time points and the relation of OTUs to specific sampling sites for (**A**) combined sources, (**B**) Nebraska Beef, (**C**) Greater Omaha and (**D**) High River-JBS. OTUs were based on abundance, including non-bacterial data (mitochondria and chloroplast). Relative abundance of organisms among specific process sampling points included Enviro/environmental samples; PM, post-marinade beef; D4, beef dried four days; D8, beef dried eight days. * Insufficient DNA was recovered for subsequent sequencing.

The bacterial diversity at the genus levels from all three processors is shown in Figure 5. Each group is an average of six samples (three samples taken from each of two separate trials) of each meat processor. The top ten most abundant genera identified were used in the relative abundance analysis. *Latilactobacillus* sp. was the dominant genus in samples taken from Nebraska Beef and High River-JBS (Figure 6A), representing 94.5% and 60.6% of the OTUs identified from each batch of samples, respectively. The highest levels of *Latilactobacillus* sp. were observed in the meat-based samples (raw beef/purge, post-marinated beef, PM; beef after four days of drying, D4; beef after eight days of drying, D8; Figure 6B,D) in which the abundance increased during processing and reached a maximum level in the day 4 samples. *Lactococcus* sp. (40.7%) and *Latilactobacillus* sp. (30.2%) were the most abundant in the samples from Greater Omaha (Figure 6C). *Lactococcus* sp. levels are initially higher compared to the *Latilactobacillus* sp. in the environmental samples and the initial raw meat/purge samples. As the meat is processed (marinated and dried), the levels of *Lactococcus* sp. decreases and levels of *Latilactobacillus* sp. increase (Figure 6D). *Lactococcus* sp. was identified in both High River-JBS and Nebraska Beef samples as well, but at less than 20% abundance in all samples. *Latilactobacillus* sp. and *Lactococcus* sp. are both lactic acid bacteria that are commonly associated with spoilage and aged beef and were expected to be in high abundance due to the use of cold-aged meat and an extended drying process [53]. Similar trends were observed using culture-dependent methodology to identify bacteria during processing.

Genera that could contain pathogenic bacteria were observed such as *Escherichia* spp. and *Pseudomonas* spp. In the environmental samples from Greater Omaha, were detected in low proportions (<1%). Additionally, low levels (<0.5%) of *Escherichia* spp. were detected in day 4 and day 8 meat samples from High River-JBS. The detection of these genera does not directly indicate the presence of a pathogenic organism in the food product. The short reads used in Illumina platform based 16S rRNA sequencing cannot be identified beyond the genus level [54]. Therefore, it is unknown if the sequence identified is pathogenic (i.e., *E. coli* O157:H7) or a non-pathogenic member of the same genus [55]. Even if it was pathogenic, the biltong process has been shown to give ≥5-log reduction to *Salmonella* serovars [1], *E. coli* O157:H7, *L. monocytogenes*, and *S. aureus* [2], and is considered sufficiently safe that USDA-FSIS does not require ingredient or end product pathogen testing if using a ‘5-log process’ [1,56].

The findings of mainly lactic acid bacteria on the raw beef samples was to be expected as they are common spoilage organisms of aged vacuum-packaged beef [39]. *Photobacterium* sp. was also identified in high proportions in the raw meat/purge samples (average of 70.8%) from High River—JBS. While commonly associated with cold marine environments, *Photobacterium* sp. have been identified in high numbers on packaged fresh beef and appear to play a role in the spoilage of meat [29,57]. As is the case with all three processors, the initial diverse communities on the raw beef gives way to a few species that become more dominant by the end of the biltong drying process. This same trend was observed in the culture-dependent data as well.

The use of a marinade during processing of a food may influence the bacterial diversity of the finished product either by contributing bacteria associated with the marinade or suppressing bacteria from the main food ingredient, allowing minor species to become more prominent. In marinated chicken breast (marinade was pH 3.7–4.2), the predominant lactic acid bacteria found were *Latilactobacillus plantarum*, *L. paracasei* subsp. *paracasei*, *L. parabuchneri*, and *L. brevis* instead of typical meat spoilage organisms, such as *L. sakei*, *L. curvatus*, and *Carnobacterium* sp. Additionally, the salt in the marinade may also contribute to the prevalence of the lactic acid bacteria that are halotolerant during processing. The marinade used during biltong processing for this study is 2.2% NaCl (*w*/*w*). In other reduced-sodium studies, sausage products with similar salt levels (2.0% *w*/*w*) that were vacuum packaged had a core community consisting of *Latilactobacillus sakei*, *Lactococcus piscium*, *C. divergens*, *C. maltaromaticum*, *Serratia proteamaculans*, and *Brochothrix thermosphacta* [58]. The high abundance of *Latilactobacillus* sp. and *L. sakei* identified in our post-process culture-dependent data is not surprising given that it is a halotolerant bacterium found in many dried meat products, and considering the final salt concentration of biltong increases to over 4% (*w*/*w*) by the end of the process [11,21,59].

**Figure 7:**
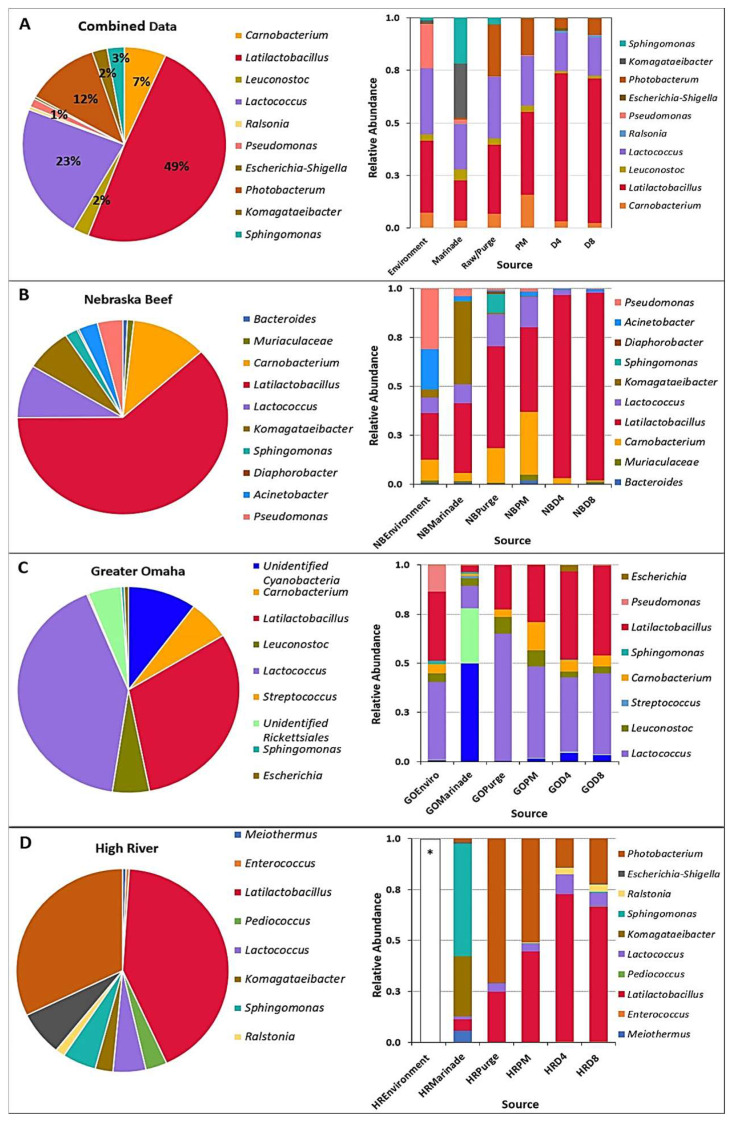
Relative abundance of total identified DNA from across all processing time points and the relation of OTUs to specific sampling sites for (**A**) combined sources, (**B**) Nebraska Beef, (**C**) Greater Omaha and (**D**) High River-JBS. OTUs were based on abundance with non-bacterial (mitochondrial and chloroplast) data removed. Relative abundance of organisms among specific process sampling points included Enviro/environmental samples; PM, post-marinade beef; D4, beef dried four days; D8, beef dried eight days. * Insufficient DNA was recovered for subsequent sequencing.

**Figure 8:**
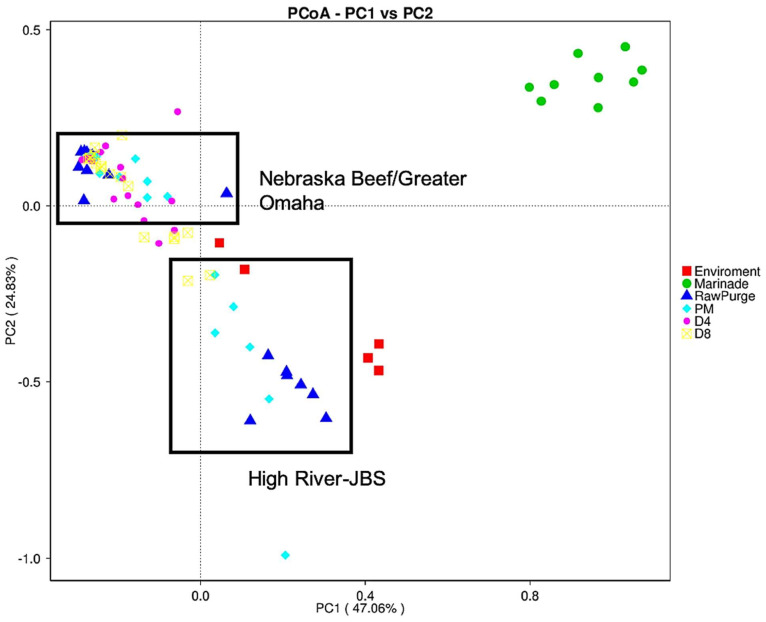
UniFrac analysis of the microbiome of all three processors combined (Nebraska Beef, Greater Omaha, and High River-JBS). Legend: the lab environment (Environment), biltong marinade (Marinade), raw meat/purge after fabrication (RawPurge), beef post-marination (PM), marinated beef dried four days (D4) and marinated beef dried eight days (D8).

There was a lack of significant differences between observed species in the individual processor data at the same drying timepoint (Figure 5). The marinade samples clustered separately from the rest of the samples. Based on the relative abundance from each processor (Figure 6), the marinade samples had a drastically different microbial community composition compared to the other meat samples, which could account for the separate clustering. The marinade was made up primarily of *Cyanobacterium*, chloroplast, and *Rickettsiales*, likely due to the marinade being made of primarily plant material (i.e., spices, such as coriander and pepper) and was present in higher proportion within the marinade samples compared to the others collected. Given that the marinade is an acidic, vinegar-based marinade, it was expected that the bacterial load in the marinade samples would be low (microbial counts drop after marination, Figure 3) and that the plant material in the marinade would yield higher levels of chloroplasts. Although the chloroplast data were not initially removed from the bioinformatic analysis, it was a small proportion of the subsequent beef samples which is the mainstay of this study (Figure 6A–D). Plant chloroplast 16S rRNA and bacterial 16S rRNA genes share high sequence similarity as they are evolutionary descendants from bacteria [60]. The universal primers targeting the 16S rRNA gene can influence non-specific binding, and given the likely low population of bacterial material available in the marinade, the primers could then bind to the chloroplast rRNA instead given the similar homology [61]. Confirmation of the minimal impact of the marinade on the meat samples was performed by subsequently removing chloroplast and mitochondrial data from the analysis (Figure 7). Distinct clustering between meat samples across all processors was also observed in the UniFrac analysis (Figure 8). The UniFrac analysis uses phylogenetic data derived from microbiome sequencing to compute genetic differences between the taxa. Our data show proximity of the microbial communities of Nebraska Beef and Greater Omaha Packing, both based out of Nebraska, while High River-JBS beef was sourced from neighboring Colorado, and the sources of animals from which the beef are harvested likely overlap (Figure 8). It was presumed there might be significant influence from environmental contamination of harvested beef from the processing facilities; perhaps seeking beef from sources that are more geographically dispersed might highlight greater differences between the taxa.

The lack of overlap of some of our culture-dependent vs. culture-independent data is not surprising as many studies have alluded to the inability of culture-based methods to recover a complete representation of organisms. We used only a single medium in our culture-dependent approach, which might explain the difference between our final processing results (8-day) with Greater Omaha sourced beef showing predominantly *Latilactobacillus* spp., and *Carnobacterium* spp., while the culture-independent approach demonstrated predominantly *Lactococcus* spp. One reason could be the recovery medium used for culture-dependent isolation was unsupportive of *Lactococcus* spp. Some investigators have examined as many as four [62] to eight [63] growth media for comparison with culture-independent methods for improved detection of OTUs. Anguita-Maeso et al. (2020) examined xylem-colonizing bacteria of olive plants and found only 41% of the total genera using culture-dependent methods as was found by culture-independent methods by Illumina MiSeq 16S rRNA sequencing using NGS [62]. Alou et al. (2021) have suggested that media supplements or additives can be used to selectively culture nondominant species that would otherwise be missed [64].

Upon removal of the non-bacterial data, a change in the total microbial profile can be seen for samples from several of the individual processors (Figure 7B,D). However, the same dominant genera from the initial analysis remain, including *Latilactobacillus* sp., *Lactococcus* sp., and *Photobacterium* sp. Further investigation of the microbial profile of the samples from each step in the biltong process (Figure 7A–D) reveals the marinade now has a different profile but still with minimal overlapping genera on the subsequent beef samples as previously seen with the analysis, including the non-bacterial data. The microbial profiles of the beef samples (purge, PM, D4, D8) are comprised of different bacteria than those observed in the marinade samples with an increase in the abundance of *Latilactobacillus* sp. by the end of biltong processing. This gives strength to the notion that the chemistry (acidic vinegar, salt) and conditions (desiccation, low A_w_) selects for those bacteria that can withstand these conditions and are noticeably present at the end of the process.

## 4. Conclusions

Raw beef from different meat processors had diverse microbial compositions. Regardless of this diversity on raw beef, the finished biltong product (day 8) was predominantly comprised of *Latilactobacillus* sp. and *Lactococcus* sp. based on relative abundance analysis of the bacterial community. The culture-dependent analyses showed mostly *Latilactobacillus sakei* and *Carnobacterium* sp. after processing. The lack of overlap of culture-dependent vs. culture-independent final data was likely due to non-ideal growth media that did not pick up the culture-independent identified abundant species.

The presence of these psychrotrophic meat spoilage bacteria is likely due to the initial vacuum-packaged refrigerated conditions (‘wet aging’, ‘vacuum-aging’) that the beef is stored in for an extended period prior to use in the biltong process. This study highlights how storage conditions of beef can influence the proliferation of psychrotrophs prior to its use in food manufacturing and how processing conditions can further cause a selective shift in the abundance of bacteria present on the final product. The culture-independent data identified *Escherichia* and *Shigella* on two of three beef sources at the end of processing (day 8), although none were recovered by our culture-dependent isolations at this stage. Although *Carnobacterium* spp. and *Latilactobacillus* spp. are often observed as spoilage organisms of raw meat products, they can also be viewed as protective non-pathogenic cultures by their ability to out-compete mesophilic pathogenic bacteria on vacuum-packaged refrigerated raw beef products. Their predominance on raw beef attributes to their residual presence after biltong processing whereby high salt and low water activity impedes the ability of all bacteria to grow. The decline of pathogenic bacteria (5-log over 8 days of drying in our prior studies) together with the survival (recovery) of *Latilactobacillus* spp., and *Carnobacterium* spp. observed here, suggests that the biltong process can produce a safe product if storage conditions maintain product integrity of low A_w_.

## Figures and Tables

**Table 1:** Bacterial isolates identified from Greater Omaha Packing Co. Inc. (GO) based on 16S rRNA gene sequencing analysis of isolates obtained from raw beef (Raw) and marinaded beef after 8 days of drying (D8) for trial 1 (R1) and trial 2 (R2).

Sample	Genus (Species)	Sequence Length (bp)	Query Coverage (%)	Percent ID
GO Raw R1A ^1,2^	*Serratia* sp.	1477	99	97.6
GO Raw R1B ^1,2^	*Carnobacterium* sp.	1411	100	98.1
GO Raw R1C ^1,2^	*Lactococcus piscium*	1449	98	99.7
GO Raw R1D ^1,2^	*Carnobacterium divergens*	1421	100	99.8
GO Raw R1E ^1,2^	*Lactococcus piscium*	1449	98	99.7
GO Raw R2A ^1,2^	*Carnobacterium divergens*	1407	100	99.9
GO Raw R2B ^1,2^	*Carnobacterium divergens*	1467	98	99.7
GO Raw R2C ^1,2^	*Carnobacterium* sp.	1420	99	98.1
GO Raw R2D ^1,2^	*Latilactobacillus sakei*	1382	100	99.9
GO Raw R2E ^1,2^	*Latilactobacillus sakei*	1408	100	100.0
GO D8Mar R1A ^1,3^	*Latilactobacillus sakei*	1489	99	99.7
GO D8Mar R1B ^1,3^	*Carnobacterium divergens*	1469	98	99.7
GO D8Mar R1C ^1,3^	*Latilactobacillus sakei*	1494	99	99.7
GO D8Mar R1D ^1,3^	*Carnobacterium divergens*	1468	98	99.8
GO D8Mar R1E ^1,3^	*Latilactobacillus sakei*	1489	99	99.7
GO D8Mar R2A ^1,3^	*Latilactobacillus sakei*	1490	98	99.9
GO D8Mar R2B ^1,3^	*Latilactobacillus sakei*	1493	98	99.9
GO D8Mar R2C ^1,3^	*Latilactobacillus sakei*	1489	98	99.8
GO D8Mar R2D ^1,3^	*Latilactobacillus sakei*	1475	98	99.8
GO D8Mar R2E-A ^1,3^	*Latilactobacillus sakei*	1489	98	99.7
GO D8Mar R2E-B ^1,3^	*Carnobacterium divergens*	1467	98	99.6

^1^ GO (Greater Omaha Packing Co. Inc. (Omaha, NE, USA); ^2^ Raw (raw beef); ^3^ D8Mar (marinaded beef, dried eight days).

**Table 2:** Bacterial isolates identified from Nebraska Beef (NB2) based on 16S rRNA gene sequencing analysis of isolates obtained from raw beef (Raw) and marinaded beef after 8 days of drying (D8) for trial 1 (R1) and trial 2 (R2).

Sample	Genus (Species)	Sequence Length (bp)	Query Coverage (%)	Percent ID
NB2 Raw R1A ^1,2^	*Latilactobacillus* sp. (*sakei*, *graminis*, or *curvatus*)	1501	100	99.7
NB2 Raw R1B ^1,2^	*Latilactobacillus* sp. (*graminis,* or *curvatus*)	1461	100	99.8
NB2 Raw R1C ^1,2^	*Hafnia paralvei*	1453	99	99.7
NB2 Raw R1D ^1,2^	*Carnobacterium gallinarum*	1436	99	98.5
NB2 Raw R1E ^1,2^	*Hafnia paralvei*	1472	99	99.6
NB2 Raw R2A ^1,2^	*Latilactobacillus curvatus*	1467	100	99.5
NB2 Raw R2B ^1,2^	*Latilactobacillus curvatus*	1467	100	99.7
NB2 Raw R2C ^1,2^	*Carnobacterium gallinarum*	1461	99	98.5
NB2 Raw R2D ^1,2^	*Enterobacter mori*	1472	100	99.3
NB2 Raw R2E ^1,2^	*Hafnia paralvei*	1473	99	99.6
NB2 D8Mar R1A ^1,3^	*Latilactobacillus* sp. (*sakei* or *curvatus*)	1473	100	99.6
NB2 D8Mar R1B ^1,3^	*Latilactobacillus* sp. (*sakei* or *curvatus*)	1503	100	99.7
NB2 D8Mar R1C ^1,3^	*Carnobacterium gallinarum*	1500	99	98.2
NB2 D8Mar R1D ^1,3^	*Carnobacterium gallinarum*	1472	100	98.2
NB2 D8Mar R1E ^1,3^	*Latilactobacillus* sp. (*sakei* or *curvatus*)	1498	100	99.7
NB2 D8Mar R2A ^1,3^	*Leuconostoc mesenteroides*	1441	100	99.4
NB2 D8Mar R2B ^1,3^	*Leuconostoc mesenteroides*	1457	100	99.9
NB2 D8Mar R2C ^1,3^	*Latilactobacillus* sp. (*sakei* or *curvatus*)	1524	100	99.7
NB2 D8Mar R2D ^1,3^	*Latilactobacillus* sp. (*sakei* or *curvatus*)	1500	100	99.6
NB2 D8Mar R2E ^1,3^	*Latilactobacillus* sp. (*sakei* or *curvatus*)	1467	100	99.7

^1^ NB2 (Nebraska Beef); ^2^ Raw (raw beef); ^3^ D8Mar (marinaded beef, dried eight days).

**Table 3:** Bacterial isolates identified from High River-JBS (HR) based on 16S rRNA gene sequencing analysis of isolates obtained from raw beef (Raw) and marinaded beef after 8 days of drying (D8) for trial 1 (R1) and trial 2 (R2).

Sample	Genus (Species)	Sequence Length (bp)	Query Coverage (%)	Percent ID
HR Raw R1A ^1,2^	*Carnobacterium divergens*	1472	98	99.5
HR Raw R1B ^1,2^	*Carnobacterium divergens*	1519	96	98.6
HR Raw R1C ^1,2^	*Carnobacterium divergens*	1440	96	100.0
HR Raw R1D ^1,2^	*Carnobacterium divergens*	1481	97	99.2
HR Raw R1E ^1,2^	*Leuconostoc mesenteroides*	1477	99	99.7
HR Raw R2A ^1,2^	*Carnobacterium divergens*	1504	96	99.3
HR Raw R2B ^1,2^	*Brevibacillus invocatus*	1467	99	98.8
HR Raw R2C ^1,2^	*Carnobacterium divergens*	1477	96	99.6
HR Raw R2D ^1,2^	*Carnobacterium divergens*	1510	96	98.2
HR Raw R2E ^1,2^	*Carnobacterium divergens*	1506	96	98.4
HR D8Mar R1A ^1,3^	*Lactilactobacillus sakei*	1494	100	99.5
HR D8Mar R1B ^1,3^	*Carnobacterium divergens*	1485	98	98.5
HR D8Mar R1C ^1,3^	*Carnobacterium divergens*	1478	98	98.8
HR D8Mar R1D ^1,3^	*Carnobacterium divergens*	1448	98	99.2
HR D8Mar R1E ^1,3^	*Carnobacterium divergens*	1441	99	99.3
HR D8Mar R2A ^1,3^	*Latilactobacillus sakei*	1512	100	99.3
HR D8Mar R2B ^1,3^	*Carnobacterium divergens*	1476	98	99.3
HR D8Mar R2C ^1,3^	*Carnobacterium divergens*	1546	96	98.9
HR D8Mar R2D ^1,3^	*Brevibacillus invocatus*	1471	99	98.9
HR D8Mar R2E ^1,3^	*Carnobacterium divergens*	1502	96	99.4

^1^ HR (High River Angus-JBS); ^2^ Raw (raw beef); ^3^ D8Mar (marinated beef, dried eight days).

## Data Availability

The data presented in this study are available on request from the corresponding author.
